# Microalgae as a Novel Therapy for Chronic Wound Healing

**DOI:** 10.1111/iwj.70887

**Published:** 2026-03-20

**Authors:** Saskia Pearl, Yang Jae Kang, Jungnam Cho

**Affiliations:** ^1^ Department of Biosciences Durham University Durham UK; ^2^ Division of Bio & Medical Bigdata, Department of Brain Korea 21 Four Gyeongsang National University Jinju Republic of Korea; ^3^ Division of Life Science Gyeongsang National University Jinju Republic of Korea; ^4^ Research Institute of Molecular Alchemy, Gyeongsang National University Jinju Republic of Korea

**Keywords:** chronic wound, hypoxia, microalgae, photosynthesis‐based oxygen delivery

## Abstract

Chronic wounds pose a substantial global health challenge, marked by persistent inflammation, infection, hypoxia, and impaired tissue regeneration. Traditional oxygen‐based therapies, including hyperbaric and topical oxygen treatments, often suffer from limited efficacy, high costs, restricted accessibility, and difficulties in achieving sustained oxygen delivery. In contrast, microalgae offer a promising and sustainable alternative, owing to their biocompatibility, glucose consumption, and continuous oxygen production via photosynthesis. Innovative delivery platforms, such as hydrogels, scaffolds, sutures, microneedles, and microrobots, have demonstrated enhanced wound healing by mitigating hypoxia, reducing infection, and modulating inflammation. Recent advances in genetic engineering and 3D bioprinting further enhance the therapeutic potential of these systems. This review explores current progress in microalgae‐based wound healing approaches, with a particular focus on photosynthesis‐driven oxygen delivery technologies.

## Short Summary

Chronic wounds present a major global health issue due to persistent inflammation, infection, and poor tissue regeneration, with traditional oxygen therapies often falling short in efficacy and accessibility. Microalgae‐based systems, enhanced by advanced delivery platforms and technologies like genetic engineering and 3D bioprinting, offer a promising, sustainable alternative through continuous photosynthetic oxygen production and improved wound healing outcomes.

## 1 Introduction

Chronic wounds are extremely prevalent worldwide and are expected to continue to increase with the rise of non‐communicable diseases such as type II diabetes [1]. These wounds, such as arterial, venous, diabetic, or pressure ulcers, are characterized by their failure to produce anatomic and functional integrity within 3 months [2, 3]. An estimated 40 million people develop chronic wounds globally, significantly impacting their quality of life [4, 5].

Wound healing involves four phases: haemostasis, inflammation, proliferation, and remodelling [6]. Haemostasis occurs immediately after injury and is characterized by fibrin clot formation and vascular constriction to prevent blood loss [7]. Platelets release cytokines and growth factors, notably vascular endothelial growth factor (VEGF), stimulating the formation of new blood cells, epithelization, and collagen deposition [8]. Once bleeding is controlled, the inflammation phase begins. Neutrophils clear cellular debris and prevent bacterial contamination by releasing proteolytic enzymes and reactive oxygen species (ROS) [9]. During proliferation, epithelial cells migrate across the wound matrix while endothelial cells support angiogenesis [10]. Fibroblast cells produce collagen, a key structural component of the extracellular matrix (ECM). Finally, the provisional matrix is remodelled into structured collagen bundles resembling normal tissue.

Chronic wounds fail to proceed through the normal phases of wound healing, and instead enter a deleterious cycle [6]. Despite varying molecular causes, chronic wounds share basic characteristics such as hypoxia and elevated levels of proinflammatory cytokines, proteases, and ROS. Excess ROS can induce oxidative stress, degrading ECM proteins and reducing fibroblast and keratinocyte function [11]. Additionally, decreased blood perfusion limits oxygen supply, reducing energy availability and hindering healing [4]. Recurrent tissue damage and bacterial activity trigger an amplified and prolonged proinflammatory response [12]. The increased metabolic activity in the wound site intensifies oxygen demand, exacerbating local hypoxia [13]. Prolonged hypoxia engenders a cascade of negative effects, including bacterial growth, excess ROS production, and impaired reepithelialization, ECM synthesis, and angiogenesis [14].

Given the crucial role oxygen plays in wound healing, it is vital to deliver oxygen to chronic wounds [15]. Hyperbaric oxygen therapy (HBOT) involves exposing the body to 100% oxygen at high pressure in a specialized chamber, increasing oxygen levels in the blood plasma and tissues while promoting angiogenesis [16, 17, 18]. Studies show mixed results, with some demonstrating efficacy while others find HBOT ineffective long‐term or unable to prevent local ischemia [19, 20]. Side effects may occur, such as visual disturbance and claustrophobia [18]. Furthermore, HBOT requires equipment and staff, which may be costly or unavailable [21].

In addition, Topical Oxygen Therapy (TOT) delivers localized oxygen to promote wound healing [21]. More affordable and versatile than HBOT, TOT can be administered at home via either low constant pressure oxygen devices using a plastic boot/chamber or through continuous delivery of non‐pressurized oxygen systems employing portable, battery‐powered generators [21]. However, limited clinical evidence and inconsistent results persist, with variable efficacy and poor gas penetration to tissues reported [21, 22, 23]. Oxygen‐releasing materials such as peroxides (e.g., calcium peroxide, sodium percarbonate) and fluorinated compounds (e.g., perfluorocarbons) can also be incorporated into biomaterials for TOT [24]. However, these materials can generate excessive ROS and release oxygen unpredictably, risking cellular damage while offering only short‐term oxygen delivery [25].

In short, established therapies aim to reduce pain, optimize wound healing, and prevent infection, with this review focusing on oxygen delivery therapies [26]. However, conventional oxygen delivery therapies have limitations relating to efficacy, convenience, and safety [27]. Current research has therefore turned towards alternative solutions, such as microalgae, photosynthetic microorganisms that live in terrestrial and aquatic habitats [28]. They produce copious amounts of oxygen through photosynthesis and can be integrated into various platforms to deliver oxygen to wounds [29]. While previous reviews have explored photosynthesis‐based oxygen therapies, few center around microalgae in wound healing [30]. This review therefore aims to highlight research on wound healing properties of microalgae and their application in photosynthesis‐based treatments.

## 2 Main Text

### 2.1 Microalgal Bioactive Compounds

Since chronic wounds contain excess ROS, harmful microorganisms, and high levels of inflammation, an exemplary wound dressing would possess antioxidant, antibacterial, and anti‐inflammatory characteristics, amongst others. The following sections explore the potential of live microalgae and its bioactive compounds in wound healing.

Microalgae produce a variety of bioactive compounds that can be integrated into synthetic (e.g., polyacrylamide, polyvinyl alcohol) or biopolymer‐based (e.g., alginate, cellulose, chitosan, gelatin) wound dressings to accelerate healing [31, 32, 33]. These exhibit crucial roles in reducing inflammation, preventing infection, and scavenging ROS (Table 1).

**TABLE 1:** Wound healing attributes of key microalgal bioactive compounds.

Bioactive compound	Species	Wound model	Chronic wound healing effect	References
β‐carotene	*Chlorella vulgaris*	animal	Increases tissue integrity and immune cell activity; antioxidant activity	[34]
*Chlorophyll a*	*Microcystis aeruginosa*	animal	Inhibits TNF‐α gene expression; anti‐inflammatory activity	[35]
Astaxanthin	*Haematococcus pluvialis*	animal	Inhibits expression of inflammatory cytokines (TNF‐α, COX‐2, IL‐6, IL‐8); promotes cell migration and angiogenesis	[36, 37]
β‐Glucans	synthetic	animal	Trigger immune cell activity; reduce elevation of inflammatory cytokines (IL‐6, VEGF)	[38]
Flavonoids & alkaloids	*Spirulina* spp.	animal	Antimicrobial activity; aids wound contraction and epithelisation	[39]
Exopolysaccharides	*Nostoc* PCC7413 & PCC7936	In vitro	Promotes fibroblast proliferation and migration	[40]
Pectin	*Spirulina maxima*	animal	Promotes cell proliferation and the upregulation of genes associated with anti‐inflammatory growth factors, cytokines, and metalloproteinases	[41]
C‐phycocyanin	*Spirulina maxima*	In vitro	Support keratinocyte migration; anti‐inflammatory and antibacterial activity	[42]

Abbreviations: COX, Cyclooxygenase; IL, interleukin; TNF‐*α*, tumour necrosis factor.

Bioactive compounds from microalgae can reduce inflammation by modulating signalling pathways, inhibiting pro‐inflammatory cytokines (TNF‐*α*, IL‐6, IL‐8) and producing enzymes involved in the scavenging of ROS, such as collagenolytic enzyme [43, 44]. Polysaccharides are particularly effective at reducing inflammation across microalgae species. A *Spirulina maxima*‐based pectin (SmP) and exopolysaccharides (EPS) from *Nostoc* sp. (strains PCC7936 and PCC7413) both demonstrated significant anti‐inflammatory effects. SmP increased in vitro human dermal fibroblast proliferation by 20%–40% compared to controls while reducing inflammatory cytokine production [41, 45]. The *Nostoc* sp. EPS also promoted fibroblast proliferation and migration while aiding the removal of inflammatory products such as bacteria, dead tissue, and accumulated neutrophils [40]. These findings align with other studies highlighting the anti‐inflammatory activity of polysaccharide extracts [46, 47, 48].

Moreover, polysaccharides display anti‐bacterial properties by disrupting biofilms, hindering bacterial adhesion, and increasing the permeability of bacterial cell membranes [49]. *Spirulina maxima‐derived* C‐phycocyanin has shown potent antibacterial activity [42]. In this study, an argon atmospheric plasma jet transformed *Spirulina maxima* biomass into viable bioactive coatings. These coatings displayed antibacterial activity against *Staphylococcus aureus* and *Pseudomonas aeruginosa* and promoted anti‐inflammatory responses [42]. Plasma‐treated *Spirulina maxima* showed 93% bacterial cell death against *Pseudomonas aeruginosa* and 73% against *Staphylococcus aureus* , compared to 82% and 35%, respectively, for untreated *Spirulina maxima;* however, the underlying antibacterial mechanisms remain to be investigated [42].

In addition to the anti‐inflammatory and anti‐bacterial effects, microalgae contain potent antioxidant compounds, including phycocyanin, chlorophyll a, polysaccharides, and carotenoids [50]. *Spirulina* polysaccharide complex (SPC) has been demonstrated to induce *Superoxide Dismutase 2* expression, a critical antioxidant enzyme, thereby reducing oxidative stress and enhancing collagen production [51]. Additionally, *Haematococcus pluvialis* cells enriched with the keto‐carotenoid astaxanthin were shown to effectively reduce oxidative stress, promote angiogenesis, and mitigate inflammation in burn wounds [36, 37]. However, clinical trials are needed to confirm the efficacy of astaxanthin in diverse wound types, apart from burn wounds. Future studies should also quantify antioxidant secretion, release profiles, and bioavailability in clinically relevant wound models to clarify the contribution of microalgal antioxidants to host ROS regulation.

### 2.2 Additional Advantages of Live Microalgae in Wound Healing

Hyperglycaemia in diabetic chronic wounds (DCWs) hinders closure by disrupting cell defence mechanisms, angiogenesis, and collagen crosslinking [52]. These elevated glucose levels increase pro‐inflammatory cytokines while reducing fibroblast activity and protein synthesis, leading to prolonged inflammation. While enzyme‐based treatments such as glucose oxidase have been explored, they face challenges including molecular instability and rapid degradation [45]. Research suggests that microalgae might offer a biological alternative. Under optimal continuous illumination (800 lx), a *Chlorella sp* solution (1 × 10^9^ cell/mL) efficiently consumed glucose in concentration‐dependent patterns [53]. Chlorella‐based hydrogels effectively reduced glucose levels in simulated wound environments, potentially addressing hyperglycaemic conditions in DCWs [53]. Results showed that light duration rather than intensity was critical in determining glucose consumption efficiency.

Biocompatibility is a crucial requirement for wound‐healing biomaterials to prevent adverse reactions [54]. Microalgae‐based treatments, particularly those containing *Chlamydomonas reinhardtii* , exhibit low cytotoxicity and do not elicit substantial immune responses or irritation. Recognized as ‘Generally Regarded as Safe’ by the FDA, *Chlamydomonas reinhardtii* has been further validated in studies showing that *Chlamydomonas reinhardtii* ‐loaded alginate hydrogels caused no irritation, pain, or burning in 17 out of 20 volunteers [55, 56]. Moreover, *Chlamydomonas reinhardtii‐based* photosynthetic scaffolds implanted in skin wounds of 8 patients showed no immune response and facilitated complete tissue regeneration over 90 days [57]. As for other microalgae, studies looking at *Chlorella vulgaris* hydrogels, *Spirulina platensis* extract incorporated skin cream, *Spirulina elongatus* encapsulated alginate hydrogel microparticles, and *Euglena gracilis* cells mixed with chitosan‐hyaluronic acid all cited excellent biocompatibility [50, 58, 59, 60].

Further to these advantages, multiple studies have demonstrated the low‐cost nature of microalgae, with a 9 cm^2^ alga‐gel patch containing *Synechoccus elongatus* costing under $1 in the laboratory [19, 61, 62]. Microalgae possess a distinctive combination of attributes from plants and microbial cells which enables sustainable biomass production, including efficient photosynthesis, rapid growth, ability to secrete metabolites, and simple extraction procedures [30, 40, 63].

In short, microalgae are advantageous for chronic wound healing due to their abundance of bioactive compounds and consumption of glucose in DCWs. These biological effects are elevated by their practical advantages such as low cost, sustainability, and biocompatibility. It is also noteworthy that only a fraction of bioactive compounds, including astaxanthin and β‐carotene, have been manufactured at an industrial scale due to challenges isolating the compounds [64]. Nonetheless, microalgal bioactive compounds undoubtedly offer substantial benefits for wound healing, and in fact, many oxygen delivery systems integrate both microalgae and their extracts for additional therapeutic benefits.

### 2.3 Photosynthesis‐Based Oxygen Delivery Methods

**FIGURE 1:**
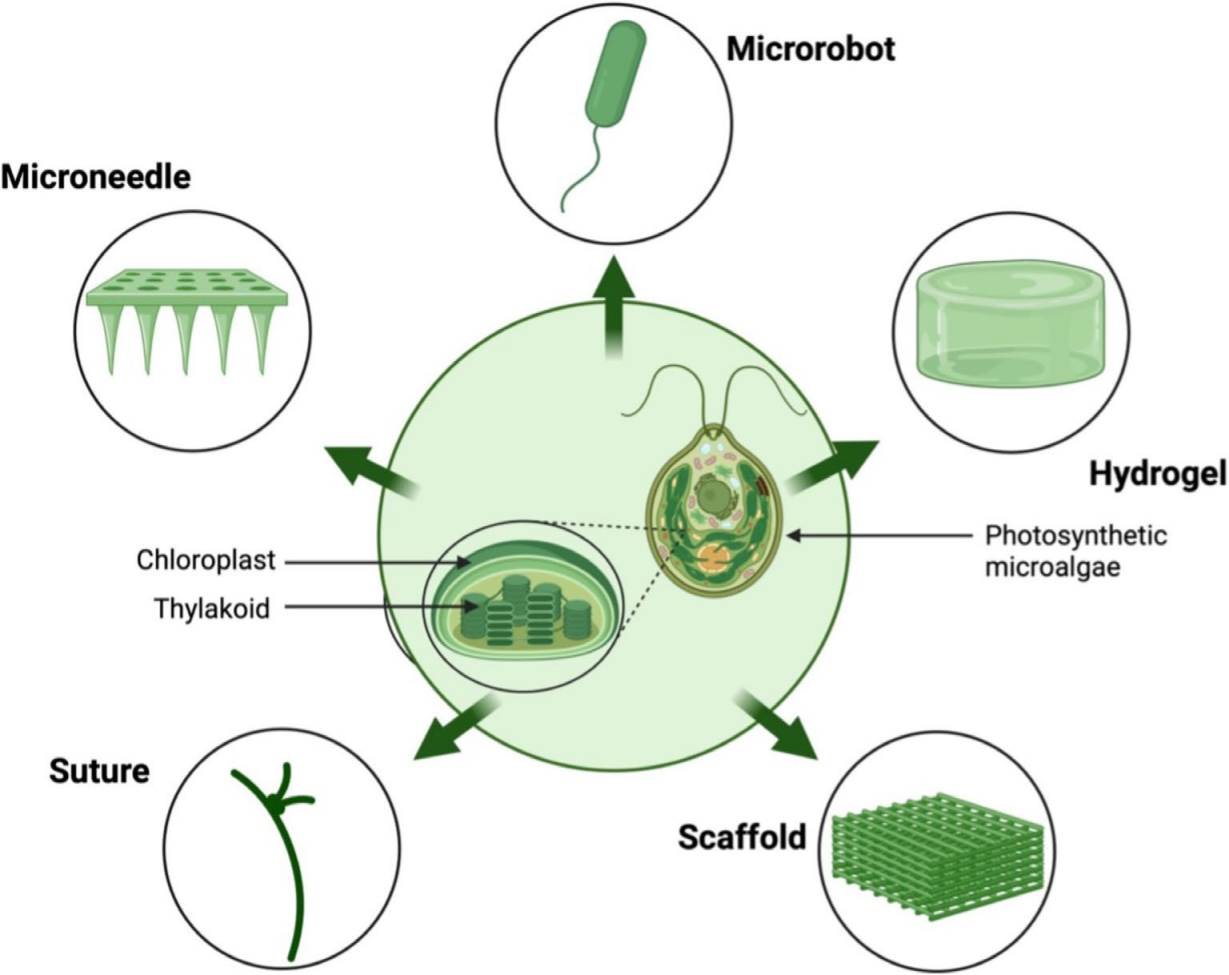
Photosynthetic‐based oxygen delivery methods.

A notable advantage of using microalgae in wound healing is their ability to continually produce oxygen. Oxygen is crucial for wound healing, and hypoxia is deemed the main cause of chronic wounds [57, 65]. Hence, in this section, five novel techniques by which oxygen is delivered to the wound will be introduced (Figure 1).

Hydrogels are 3D networks of cross‐linked hydrophilic polymers that retain considerable amounts of water and biological fluids [36]. They are durable, chemically and physically tunable, and injectable, making them an attractive option for wound healing dressings [66, 67]. Multiple studies incorporating photosynthetic microalgae into hydrogels have demonstrated sustained oxygen production [49, 58, 68, 69]. Alginate hydrogels loaded with *Chlamydomonas reinhardtii* showed immediate oxygen release when illuminated and maintained a high oxygen release capacity for 7 days [56]. Similarly, a photo‐crosslinked *Chlamydomonas reinhardtii* carboxymethyl chitosan continuously photosynthesized under illumination while displaying antibacterial properties due to the incorporation of ciprofloxacin antibiotic [67]. *Synechoccus elongatus* (strain PCC7942) incorporated into algal gel patches also produced sustained oxygen, with the hydrophilic polytetrafluoroethylene film creating a localized moist environment with high oxygen levels to deliver dissolved oxygen to the wound [19]. Furthermore, oxygen‐generating hydrogels alleviate inflammation, infection risk, and hypoxia, while promoting angiogenesis and cell proliferation in mouse models [70]. Therefore, they consistently show superior wound closure rates compared to controls. The ciprofloxacin‐integrated *Chlamydomonas reinhardtii* hydrogels in diabetic mice achieved 96.70% wound closure by day 12, compared to 78.98% in controls [67]. *Synechoccus elongatus* hydrogels achieved 45% wound closure by day 6, representing over 100‐fold greater efficiency than conventional TOT [19]. Nevertheless, the short duration of all studies (2–3 weeks) and the use of mouse models restrict the assessment of long‐term efficacy and clinical relevance in humans. Additionally, most hydrogels only penetrate surface layers and may not effectively oxygenate deep wounds. Future research should investigate hydrogels that can improve depth penetration and maintain stability across patient populations and wound types.

Scaffolds provide a structural framework and offer more mechanical stability than hydrogels. Clinical translation has progressed the furthest with scaffolds, with one study evaluating the safety and feasibility of photosynthetic scaffolds in 8 human patients with full‐thickness skin wounds [57]. *Chlamydomonas reinhardtii* scaffolds were surgically implanted into patients and illuminated with a device that employed light/dark cycles to promote photosynthesis [57]. Over 90 days, macroscopic analyses showed no immune responses, inflammation, or adverse effects, and full tissue regeneration occurred [57]. This is the first long‐term clinical trial, with 1 patient followed for 17 months showing no systemic or local side effects [55]. However, the absence of a control group limits the ability to assess the scaffolds' efficacy, and the follow up sample size of 1 restricts comparisons [55]. Further research is required to validate these findings in a larger population of patients using suitable controls. Moreover, unlike established hydrocolloid and foam dressings, photosynthetic hydrogels and scaffolds require illumination to function. Thus, while they offer a controlled oxygen supply, their practicality in clinical settings requires validation. Particularly, future studies should focus on systematically assessing species‐specific thermal tolerance, photosynthetic efficiency, and metabolite release in clinically relevant wound environments.

Photosynthetic systems can also be integrated with other specialized carriers, such as sutures, to deliver oxygen. Traditional surgical sutures serve a mechanical role in wound closure, lacking biological functionality [71]. However, genetically engineered *Chlamydomonas reinhardtii* (strains UVM4‐GFP and UVM4‐VEGF) were incorporated into sutures to promote vascularization, oxygen release, stem cell recruitment, and the secretion of recombinant VEGF, platelet‐derived growth factor (PDGF), and stromal derived factor 1 (SDF‐1α) [72]. Polyglactin sutures were seeded with photomixotrophically grown *Chlamydomonas reinhardtii* [72]. Results indicated that VEGF secretion followed an exponential curve, whereas SDF‐1α secretion was inconsistent. Oxygen release was significantly higher from day 1 to 10 and plateaued from day 10 to 14. The sutures retained functional integrity for 14 days, aligning with standard suture removal timelines. Although these reflect promising preliminary results, photosynthetic sutures remain understudied compared to hydrogels and scaffolds. Of the two studies published, only one focuses on oxygen delivery, and thus more studies are needed to confirm in vivo validation [71, 72].

Since hydrogel systems are based on passive diffusion of molecules to the wound, they risk being blocked by barriers such as blood clots [73]. Traditional therapies, such as sutures, could lead to local mechanical stress and ultimately cause scars [62]. Microneedles (MNs) have been developed to overcome these limitations [74]. Composed of micron‐sized fine tips arranged on a base, MNs can penetrate blood clots, allowing for precise and targeted drug delivery directly to the wound [25]. Smart MNs incorporate drug‐loaded biomaterials that react to physiological or biochemical triggers, enabling controlled and responsive drug release [75]. The first study that examined the potential of MNs to encapsulate live organisms produced a *Chlorella vulgaris* loaded separable MN (CvMN), consisting of gelatin methacryloyl (GelMa) tips and a polyvinyl acetate (PVA) substrate [62]. Upon application to diabetic mice wounds (*n* = 6), the PVA layer dissolved, leaving the GelMa tips embedded in the skin. Under NIR LED light, *Chlorella vulgaris* generated a steady supply of oxygen, enhancing fibroblast proliferation and angiogenesis [62]. *Chlorella vulgaris* remained viable for over 6 days, ensuring sustained oxygen release. Both in vitro and in vivo studies confirmed that CvMN reduced inflammation and accelerated wound closure [62]. Following this, a different study developed *Chlorella*‐loaded poly (ionic liquid)‐based MNs (PILMN‐Chl) for treating methicillin‐resistant *Staphylococcus aureus* infected DCWs [25]. The MNs continuously produced oxygen for over 30 h, reducing hypoxia while eliminating over 90% of bacteria. PILMN‐Chl also promoted fibroblast proliferation, angiogenesis, and collagen deposition, accelerating DCW healing in mice [25]. It is, however, worth noting that MNs have more general limitations, such as restricted drug doses and challenges with vertical penetration, which reduce their effectiveness in penetrating irregular wound surfaces [62]. Furthermore, MNs have limitations pertaining to skin irritation and the immune response, as well as risk of infection from leftover MN tips in wounds [73]. Nevertheless, these results represent a promising treatment option and further studies are needed to explore microalgal‐based MNs in human wounds.

Given the limitations of MNs related to skin irritation and immune responses, biohybrid microrobots have been proposed to noninvasively deliver oxygen through blood clots [73, 76]. These robots use self‐propelling motors, taking advantage of the small size of microalgae (10 nm–10 μm) and their unique natural motility capabilities [77]. One study developed *Chlamydomonas reinhardtii* ‐based microrobots coated with a chitosan‐heparin nanocomplex [73]. The robot moved autonomously at 33.3 μm s^−1^ using self‐propulsion via photosynthesis and the swimming motion from flagella. Wounds treated with the microrobot were completely healed within 12 days, outperforming control groups. Furthermore, the microrobots efficiently penetrated through medium density blood clots, produced enough oxygen to alleviate hypoxia, and scavenged inflammatory chemokines, reducing inflammatory cytokine levels by up to 31% [73]. Histological analysis revealed enhanced collagen deposition and angiogenesis in microrobot‐treated wounds. The study employed robust methodologies, including short term in vivo and in vitro tests on mice [73].

Taken together, these approaches show potential by reducing hypoxia, infection risk, and inflammation while promoting angiogenesis. Oxygen penetration depth varies considerably, ranging from shallow (hydrogels and scaffolds) to deep tissue (microrobots). Hydrogels and scaffolds appear most suitable for surface wounds, while microneedles and microrobots show greater promise for deep wounds requiring targeted oxygen delivery. Clinical translation remains the predominant challenge, with only scaffolds reaching clinical trial stages and hydrogels likely next due to their thorough preclinical validation. Microneedles, sutures, and microrobots remain understudied, highlighting the need for future research.

### 2.4 Challenges of Microalgal Photosynthesis‐Based Treatments

Although the preceding sections have demonstrated the significant potential of microalgae in wound healing, microalgal treatments face challenges. These include technical barriers such as light delivery and clinical translation obstacles. The following section addresses these challenges while exploring innovative approaches.

A crucial prerequisite for photosynthesis is sufficient light supply, which is determined by factors such as light intensity, irradiation time, irradiation position, and wavelength [29]. Although light supply is plentiful for exposed wounds, current in vivo illumination techniques are unable to provide light at great depths nor meet wavelength and power demands, significantly limiting photosynthesis [78]. Directly delivering light sources inside the body is an encouraging solution, with a wireless light‐emitting device shown to be capable of safe and precise deep‐tissue penetration [79]. In this study, a flexible tapered optical fibre was embedded in a transparent polyester patch, aiming to improve light dispersion by enhancing optical energy scattering [79]. Although its effectiveness was constrained by limited energy conversion efficiency and biocompatibility challenges, it reflects advances in implantable light delivery device research [79]. Therefore, the limitation of light penetration may soon be overcome, potentially enabling oxygen delivery to previously inaccessible deep wounds [80]. Future studies should also systematically evaluate microalgal survival under clinically relevant wound conditions, including inflammatory stress and nutrient limitation, and optimize scaffold design, illumination parameters, and dosing strategies accordingly.

Although laboratory and sparse clinical studies have shown positive wound healing results, the efficacy of some microalgal therapies may not exceed that of established wound healing methods. Comparative studies to assess their effectiveness relative to traditional approaches and to identify limitations that may impede clinical applications are necessary [56]. Clinical outcomes will inevitably vary between individuals and wound types, and therefore studies determining efficacy should optimize factors such as illumination settings and microalgae density while accounting for injury type [57]. Safety levels likely differ between microalgal species, with *Chlamydomonas reinhardtii* 's FDA ‘Generally Regarded as Safe’ status offering a clearer regulatory pathway than less‐studied species that may require more extensive biocompatibility evaluation. Additionally, research is primarily limited to the backs of animal models, which experience minimal movement. However, these wounds differ markedly from tension wounds at joints, highlighting the need for studies that better replicate clinical conditions. Beyond clinical applicability, long‐term biosafety is a critical concern, as secondary metabolites produced by living microalgae at the wound site could pose unknown risks [81]. Experiments should monitor pain, erythema, swelling, and reactions to algae in peripheral tissues long‐term (over 12 weeks). In the future, establishing standardized efficacy metrics and conducting larger clinical trials comparing microalgal therapies with conventional treatments will help determine their clinical utility.

## 3 Conclusion and Future Perspectives

This review has explored the current state of microalgae‐driven approaches for wound healing, with a particular focus on emerging photosynthesis‐based oxygen delivery systems. The field has rapidly evolved since 2020, and studies reveal clear potential across delivery platforms. Both microalgae and their bioactive compounds alleviate hypoxia, inflammation, impaired tissue regeneration, and bacterial contamination. Hydrogels and scaffolds have been the most extensively studied, while more novel techniques such as sutures, microrobots, and MNs have only been verified by a few studies. These treatments show particular promise for DCWs by addressing central pathological features, such as hypoxia and hyperglycaemia. Furthermore, genetic engineering and 3D bioprinting approaches offer potential for optimizing microalgal properties. However, most studies are short term (2–3 weeks), conducted in murine models with artificially created wounds, and lack standardized assessment metrics, making direct efficacy comparisons challenging. Comparative analyses between photosynthesis‐based delivery methods are complicated by variations in microalgae species, illumination parameters, and wound types. Technical challenges, such as light penetration limitations, also persist. Future research must advance beyond laboratory studies towards standardized efficacy metrics, long‐term safety studies, and larger human clinical trials on diverse wound types. If these challenges are addressed, microalgal photosynthesis‐based therapies could revolutionize chronic wound care by providing an accessible, sustainable, and effective solution.

Genetically engineered microalgae have potential in wound healing by simultaneously delivering oxygen and bioactive molecules [57]. Advances in CRISPR‐Cas9 have enabled precise, low‐cost, and efficient gene editing to become increasingly more accessible. As previously mentioned, *Chlamydomonas reinhardtii* and *Spirulina platensis* have been genetically modified to secrete growth factors such as VEGF, PDGF, and SDF‐1*α*, promoting angiogenesis, cell migration, and tissue regeneration [72]. A subsequent study developed *Chlamydomonas reinhardtii* strains that exhibited enhanced expression and secretion of the human growth factors hVEGF‐165 [82]. However, regulatory hurdles hinder clinical translation, as genetically modified organisms require strict approval, and reduced public acceptance obstructs commercial development [83]. Unlike conventional wound treatments, these therapies may require additional biosafety approvals. Additionally, technical challenges in genome editing may slow clinical applications, such as low homologous recombination efficiency, off‐target mutations, and variable gene expression [82, 84]. Nonetheless, genetically engineered microalgae have significant potential for wound treatments, offering a multifunctional therapy.

Emerging microfluidic bioprinting techniques allow for hydrogels to be modelled into structured 3D scaffolds with precise spatial control. When combined with microalgae and illuminated, they produce oxygen sustainably [78, 85, 86]. A Chinese herbal medicine scaffold incorporating *Chlorella pyrenoidosa* and *Panax notoginseng* saponin (PNS) was developed using a coaxial capillary microfluidic chip and an extrusion‐based 3D printer [85]. The Ca^2+^‐induced crosslinking of alginate and UV crosslinking of GelMA formed hollow microfibers printed layer by layer into porous scaffolds, facilitating oxygen delivery while promoting angiogenesis [85]. The encapsulated PNS was slowly released from the scaffold to facilitate cell adhesion, migration, proliferation, and angiogenesis. Meanwhile, the living algae produced oxygen continuously when illuminated, reducing local hypoxia [85]. Although the study lacked immunological profiling to assess the broader immune response to the scaffolds, it represents a significant step towards microalgal dressings using 3D printing technologies. As these technologies mature, customized wound dressings with precise control over microalgal density, bioactive compound release, and mechanical properties in response to environmental stimuli may be developed.

## Funding

This work was supported by the National Research Foundation of Korea (RS‐2024‐00336161 to YJK) and UKRI‐BBSRC (UKRI1915 to JC).

## Ethics Statement

The authors have nothing to report.

## Conflicts of Interest

The authors declare no conflicts of interest.

## Data Availability

Data sharing not applicable to this article as no datasets were generated or analysed during the current study.
